# The Respiratory Microbiome in Paediatric Chronic Wet Cough: What Is Known and Future Directions

**DOI:** 10.3390/jcm13010171

**Published:** 2023-12-28

**Authors:** Brianna Atto, Yitayal Anteneh, Seweryn Bialasiewicz, Michael J. Binks, Mostafa Hashemi, Jane Hill, Ruth B. Thornton, Jacob Westaway, Robyn L. Marsh

**Affiliations:** 1 School of Health Sciences, University of Tasmania, Launceston, TAS 7248, Australia; robyn.marsh@menzies.edu.au; 2 Child and Maternal Health Division, Menzies School of Health Research, Charles Darwin University, Darwin, NT 0811, Australia; yitayal.anteneh@menzies.edu.au (Y.A.); michael.binks@menzies.edu.au (M.J.B.); jacob.westaway@menzies.edu.au (J.W.); 3 Australian Centre for Ecogenomics, School of Chemistry and Molecular Biosciences, The University of Queensland, St. Lucia, QLD 4072, Australia; seweryn@uq.edu.au; 4 SAHMRI Women and Kids, South Australian Health and Medical Research Institute, Adelaide, SA 5000, Australia; 5 Department of Chemical and Biological Engineering, The University of British Columbia, Vancouver, BC V6T 1Z3, Canada; mostafai@mail.ubc.ca (M.H.); jane.hill@ubc.ca (J.H.); 6 Spire Health Technology, PBC, Seattle, WA 98195, USA; 7 Centre for Child Health Research, University of Western Australia, Perth, WA 6009, Australia; ruth.thornton@uwa.edu.au; 8 Wesfarmers Centre of Vaccines and Infectious Diseases, Telethon Kids Institute, Perth, WA 6009, Australia; 9 Centre for Tropical Bioinformatics and Molecular Biology, James Cook University, Cairns, QLD 4811, Australia

**Keywords:** paediatric, chronic suppurative lung disease, protracted bacterial bronchitis, bronchiectasis, microbiome, biofilm, microbial interactions, treatments, acute lower respiratory infection, endotyping

## Abstract

Chronic wet cough for longer than 4 weeks is a hallmark of chronic suppurative lung diseases (CSLD), including protracted bacterial bronchitis (PBB), and bronchiectasis in children. Severe lower respiratory infection early in life is a major risk factor of PBB and paediatric bronchiectasis. In these conditions, failure to clear an underlying endobronchial infection is hypothesised to drive ongoing inflammation and progressive tissue damage that culminates in irreversible bronchiectasis. Historically, the microbiology of paediatric chronic wet cough has been defined by culture-based studies focused on the detection and eradication of specific bacterial pathogens. Various ‘omics technologies now allow for a more nuanced investigation of respiratory pathobiology and are enabling development of endotype-based models of care. Recent years have seen substantial advances in defining respiratory endotypes among adults with CSLD; however, less is understood about diseases affecting children. In this review, we explore the current understanding of the airway microbiome among children with chronic wet cough related to the PBB–bronchiectasis diagnostic continuum. We explore concepts emerging from the gut–lung axis and multi-omic studies that are expected to influence PBB and bronchiectasis endotyping efforts. We also consider how our evolving understanding of the airway microbiome is translating to new approaches in chronic wet cough diagnostics and treatments.

## 1. Introduction

**Figure 1:**
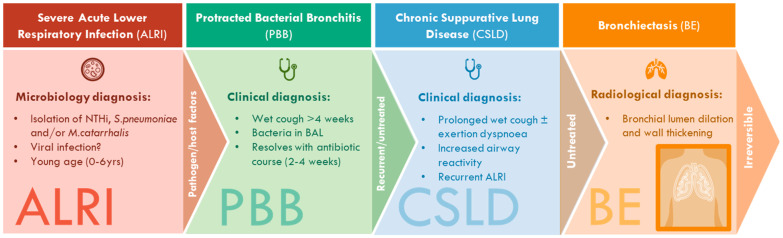
**Schema of the PBB–bronchiectasis disease spectrum and clinical definitions.** Severe acute lower respiratory infections (ALRI) early in life are associated with development of protracted bacterial bronchitis (PBB). PBB, chronic suppurative lung disease (CSLD), and radiographic-confirmed bronchiectasis share common clinical features but are different, overlapping diagnostic entities within a disease spectrum. All share the underlying mechanisms of airway neutrophilia, endobronchial bacterial infection, and impaired mucociliary clearance. ALRI is associated with an increased risk of developing PBB and, if left untreated, progression to PBB and bronchiectasis among a subset of patients. Early diagnosis and treatment are essential to preventing progression to bronchiectasis, which is initially reversible.

Cough is one of the most common reasons for paediatric medical consults worldwide [1]. Chronic wet cough for longer than 4 weeks is a hallmark of chronic suppurative lung diseases (CSLD), including recurrent protracted bacterial bronchitis (PBB) and bronchiectasis (Table 1) [2,3]. In children, PBB and bronchiectasis manifest across a diagnostic continuum, whereby persistent cough after severe acute lower respiratory infection (ALRI) is associated with increased risk of PBB [4,5], and recurrent PBB is associated with increased risk of post-infective bronchiectasis [6] (Figure 1). In hospital-based studies, 20% of children presenting with ALRI had persistent cough at their 28 day follow-up [4]; 44% of children with PBB experienced > 3 recurrent episodes in the year following diagnosis [6]; and 10% of children with PBB progressed to bronchiectasis within 5 years [7]. PBB and bronchiectasis are associated with reduced quality of life (for children and their carers) [8] and considerable healthcare costs [9]. Children with bronchiectasis experience significant morbidity and, if untreated, have poorer clinical outcomes later in life compared to patients with adult-onset bronchiectasis [10]. Thus, early recognition and prompt treatment of paediatric chronic wet cough is vital to maintaining lung health across the life course [11]; especially as bronchiectatic changes can be reversible if diagnosed early and effectively managed [12].

**Figure 2:**
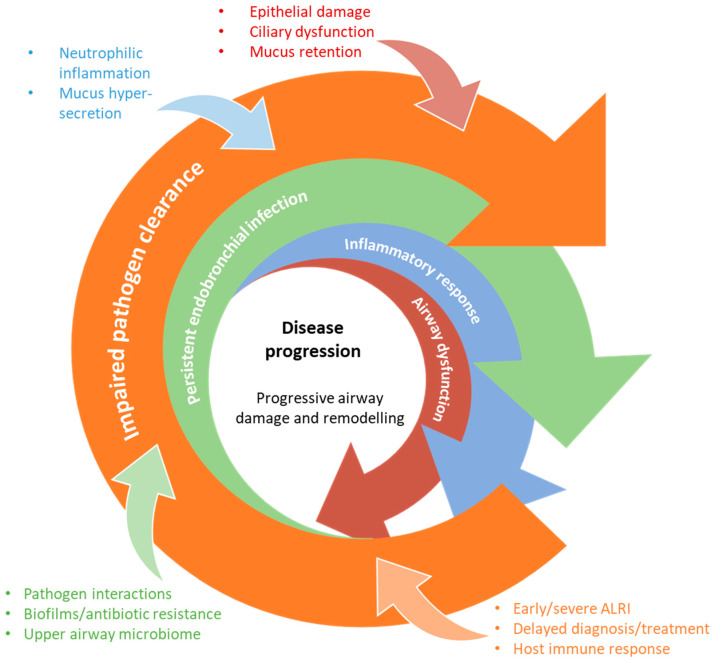
**The “vicious vortex” pathogenesis hypothesis of CSLD.** Adapted from the model originally proposed by Flume and colleagues [21]. Following an initial trigger event, host- and pathogen-associated factors interact in a non-linear fashion to drive disease progression. Chronic and progressive neutrophilic airway inflammation and mucus hypersecretion/hyper-responsiveness leads to destruction of the bronchial wall, loss of cilia, and subsequent mucociliary dysfunction and mucus retention. This impacts on pathogen clearance, resulting in a self-perpetuating cycle of endobronchial infection, inflammation, and ongoing tissue damage.

Currently, PBB and bronchiectasis management is limited by poor responsiveness of some patients to recommended treatments [3,13]. The reasons for differential treatment responses remain poorly understood, prompting ongoing research into the underlying drivers of disease onset and progression. PBB and bronchiectasis pathogenesis is hypothesised to be driven by exaggerated and/or dysregulated inflammation that is triggered by persistent endobronchial infection [14,15,16]. Failure to resolve the lower airway infection and/or the associated inflammation following an ALRI risks the establishment of persistent endobronchial infection and subsequent progression to PBB, CSLD, and bronchiectasis (Figure 1). Current pathogenesis models theorise that an initial bacterial endobronchial infection leads to neutrophilic inflammation and impaired airway clearance that results in airway damage, further growth and spread of the bacteria, and eventual establishment of chronic infection [17]. A self-perpetuating cycle of infection, inflammation, and tissue damage ensues, resulting in progressive tissue destruction that culminates in bronchiectasis. This “vicious cycle” hypothesis was first described as a model of cystic fibrosis (CF) pathogenesis in 1986 [18] and was subsequently adopted to non-CF bronchiectasis [19]. In recent years, a more nuanced model has emerged that instead describes a “vicious vortex” of host and microbial aetiological drivers that interact in a non-linear fashion (Figure 2) [20,21]. This model emphasises a complexity of factors that may differentially influence disease progression and highlights a need for individualised models of care to improve clinical outcomes [20].

The clinical complexity and disparity of treatment response among children with CSLD is suggestive of endotypes (heterogenous patient groups defined by distinct functional or pathobiological mechanisms) [21]. Characterisation of respiratory endotypes is likely to be essential to realising individualised management of PBB and bronchiectasis. Endotyping methods aim to identify pathobiological markers that can be used diagnostically or prognostically to guide specific application of current therapies or through the development of novel endotype-specific interventions [21,22]. The emergence of endotyping approaches has been supported by the ongoing advances of ‘omics technologies (e.g., genomics, transcriptomics, microbiomics, metabolomics) that enable more detailed characterisation of pathobiological markers. To date, most ‘omics-based PBB and bronchiectasis studies have been limited to measures of airway bacterial community composition [22]. Much remains to be determined about the wider airway microbiome composition, functions, and interactions with mucosal and systemic immune responses.

In this review, we discuss the current understanding of the airway microbiome among children with chronic wet cough related to the PBB–bronchiectasis diagnostic continuum, including consideration of biofilm and microbial interactions. We explore concepts emerging from the gut–lung axis and multi-‘omic studies that are expected to influence PBB and bronchiectasis endotyping efforts. Finally, we look to the future by considering how elucidating the airway microbiome is translating to new approaches in chronic wet cough diagnostics and treatments. 

## 2. The Lower Airway Microbiome among Children with PBB or Bronchiectasis

### 2.1. Bacteriome

The term “microbiome” refers to all microbes present in an environment, including bacteria, viruses, fungi, and yeasts. Although this term is commonly used in respiratory publications, most studies to date have used amplicon sequencing methods that target the bacteriome only. Species within the bacteriome are traditionally partitioned as either “pathogen” or “commensal”; however, the utility of these terms is limited given that respiratory pathogens commonly colonise the airways asymptomatically and that there is growing evidence that some commensal species may have pathobiological roles [23]. In light of this, the term “pathobiont” is used to describe potentially pathogenic species within the microbiome that only contribute to disease under certain conditions.

In culture-based studies, the pathobionts most commonly isolated from children with PBB or bronchiectasis are non-typeable *Haemophilus influenzae* (NTHi), *Streptococcus pneumoniae*, and *Moraxella catarrhalis* [3,24,25]. Several studies have reported that culture-based detection of these species is associated with the establishment and progression of PBB and bronchiectasis. For example, *M. catarrhalis* carriage was associated with persistent cough after an ALRI presentation [5], and lower airway NTHi infection was associated with a seven-fold increased risk of progression from PBB to bronchiectasis within 2 years [6]. Clinical studies have also established a relationship between reduced lung function and isolation of NTHi, *S. pneumoniae*, and/or *M. catarrhalis* [26].

Less is understood about the wider airway bacteriome as data are scarce, likely reflecting difficulties inherent in sampling the lower airway of infants and young children who do not expectorate (noting bronchoscopic sampling is only performed when clinically indicated). Additionally, published studies to date are limited by small sample sizes (typically fewer than 30 children), and methodological heterogeneity restricts interstudy comparisons [27]. Currently, only two studies have compared the airway bacteriome of children with PBB to control children [28,29]. Both studies reported significant differences in beta diversity (indicating that the PBB and control bacteriomes were different); however, alpha diversity (bacterial community structure) findings were inconsistent. Similarly, the two studies comparing airway bacteriomes of children with bronchiectasis to controls both reported no alpha diversity differences, whereas beta diversity findings were inconsistent [30,31]. Interstudy inconsistency in beta diversity is also evident across the two studies that compared PBB and bronchiectasis bacteriomes [30,32]. Low reproducibility of diversity measures across PBB and bronchiectasis studies to date likely reflects limitations of small sample sizes and effects related to heterogeneity in age, specimen type (e.g., bronchoalveolar lavage, sputum, protected brush), geographic location, and variable disease burdens among the populations studied.

Despite this variability, a small number of genera are consistently observed to be among the most abundant taxa in the airway bacteriome of children with PBB or bronchiectasis, specifically: *Haemophilus*, *Moraxella*, *Streptococci*, and *Neisseria* [28,29,30,31,32,33]. Anaerobic taxa (e.g., *Prevotella*, *Fusobacterium*, and *Veillonella*) are also common, but with higher variability in relative abundance across studies [28,29,31]. Additionally, three broad categories of bacterial community structure (alpha diversity) are common across PBB and bronchiectasis studies: (1) bacteriomes dominated by one (or sometimes two) operational taxonomic units (OTUs)/amplicon sequence variants (ASVs) that are suggestive of a recognised respiratory pathobiont (e.g., *Haemophilus*, *Moraxella*); (2) mixed profiles that include multiple taxa suggestive of oral flora; and (3) very low bacterial load bacteriomes that fail 16S rRNA gene amplification [28,29,31]. These different bacterial community structures can occur across diagnoses (including controls) [28], and manifest with high inter-individual variation within diagnostic groups [31]. Relationships between these different community structures and disease processes remain poorly understood; however, cluster-specific relationships with inflammatory endotypes have been observed among adults with bronchiectasis [34] and are suggested by the clustering observed among children with PBB [29]. While profiles dominated by *Haemophilus*, *Moraxella*, or *Streptococcus* strongly suggest infection caused by recognised pathobionts, uncertainty about species-level identification from short-read data limits interpretation. Mixed profiles composed of *Neisseria* and anaerobic taxa strongly suggest oral flora; however, it is unclear whether their presence indicates established lower airway colonisation (as suggested by data from healthy adults [35]), transient colonisation following microaspiration of oral secretions, or specimen contamination during sampling [36]. Very-low-bacterial-load communities are typically excluded from bacteriome analyses after failing to amplify or where insufficient reads are generated; however, low bacterial load is expected in the absence of infection [35]. It is unclear whether within-group heterogeneity in bacterial community structure has any relationship with the variable clinical outcomes observed among children with PBB or bronchiectasis; however, low alpha diversity (suggestive of a single OTU or ASV dominating) is associated with poorer clinical outcomes in adult bronchiectasis [34,37].

Longitudinal studies will be essential to understanding relationships between the airway bacteriome and clinical outcomes. To date, only one study has assessed the lower airway bacteriome among children with bronchiectasis longitudinally. Broderick and colleagues [33] examined the sputum bacteriome among 30 children with bronchiectasis (median age 6 years; range 1–15 years) before and after antibiotic treatment for an acute exacerbation. Despite heterogeneity in the types of antibiotics used, no significant differences in alpha or beta diversity were observed, suggesting stability of the sputum bacteriome during treatment. Longitudinal data for children with PBB are lacking.

### 2.2. Virome 

Viromes are characterised using metagenomic and metatranscriptomic methods, whereby all DNA/RNA in a specimen is sequenced. This includes human DNA, which can dominate respiratory metagenome data, even when selective depletion of host DNA is performed [38]. While metagenomic studies of adult bronchiectasis are emerging [23], we are unaware of any paediatric studies to date. As a result, current understanding of the respiratory virome among children with PBB or bronchiectasis is based only on PCR data [39,40,41,42,43,44,45,46,47].

Respiratory viruses are commonly detected from paediatric airway specimens [5,48]; however, it is unclear whether viral detection rates differ between control groups and children with PBB or bronchiectasis [25,46]. Adenovirus [41,47,49,50] and human rhinovirus (HRV) [5,25,47,51] are the most commonly reported agents in paediatric PBB and bronchiectasis; however, these findings may be dependent on population and study design [41]. Among 245 Australian children with PBB or bronchiectasis, adenovirus species C was the most commonly detected respiratory virus in BAL [47]. Higher detection rates were observed among children with PBB (20.7%) compared to those with bronchiectasis (8.1%), and adenovirus genotypes 1 and 2 predominated [47]. Of note, young age appeared to be a risk factor for lower airway adenovirus infection and was implicated in increased likelihood of bacterial co-infection [47]. Adenovirus may also play a role in long-term clinical outcomes, with bronchiectasis cases reported to increase proportionally as a function of time since acute adenovirus infection [51]. This observation is supported by data demonstrating adenovirus persistence within mucosal T lymphocytes isolated from chronically inflamed lungs [49], and positive associations with neutrophilia [25]. 

Virus presence is also common during paediatric bronchiectasis exacerbations. Kapur and colleagues [40] detected a respiratory virus (predominantly HRV [54%]) in nasopharyngeal aspirates from 48% of 69 children who were sampled during an acute bronchiectasis exacerbation. Notably, virus-positive children were more likely to require hospitalisation [40]. Despite the high HRV prevalence in the paediatric PBB and bronchiectasis populations, observed associations with disease may be confounded by the virus’ high prevalence in both symptomatic and asymptomatic childhood respiratory infections [5,39,43,52]. Thus, cautious consideration of HRV associations with disease is needed, particularly where testing is performed using upper respiratory specimens. 

There are limited data on the role of other “classic” respiratory viruses (e.g., influenzae A and B; human metapneumovirus, parainfluenzae 1, 2, and 3; and respiratory syncytial virus [RSV]), with data from some studies suggesting infrequent involvement of these viruses in PBB and bronchiectasis [40,41,47]. Similarly, there are few data on non-classical respiratory and recently described viruses (e.g., human bocavirus, human polyomavirus WU and KI, anellovirus) [40,42,46,53] and their relationship with PBB and bronchiectasis, although one study reported an association between anelloviruses and bronchiectasis [54]. Data describing the impact of SARS-CoV-2 on PBB and bronchiectasis are sparse; however, several studies found bronchiectasis can be a long-term complication following hospitalisation for COVID-19, both among adults (up to 38%) [55,56] and among children (6%) [57]. Other data suggests human T-cell lymphotropic virus type 1 is associated with bronchiectasis [58]; however, more recent research demonstrates this association does not hold for children, even when living in the same communities as affected adults [41]. Finally, an entirely different class of viruses, those that infect bacteria (bacteriophages or simply “phages”), have not yet been investigated for potential contributions to mucosal immunity and lung microbial homeostasis [59], and by extension, their role in preventing or promoting PBB and bronchiectasis. 

### 2.3. Mycobiome

There is growing interest in understanding the mycobiome (fungal and/or yeast communities) in chronic respiratory diseases, including several studies that examined the mycobiome among adults with bronchiectasis (reviewed by Martins and colleagues [22]). It remains to be seen whether an airway mycobiome contributes to paediatric PBB and bronchiectasis, as there have been no DNA-based studies to date. Culture-based data suggest a lower airway mycobiome may be less important in paediatric compared to adult bronchiectasis. In a study of BAL from 113 children newly diagnosed with bronchiectasis, all specimens were negative for fungi and yeasts by microscopic and culture-based tests [60]. 

## 3. Biofilm 

Microbiome sequencing methods assess the composition and genetic functional capacity of microbial communities; however, it is equally important that microbial phenotypes are considered when developing endotyping models. Perhaps one of the most important bacterial phenotypes requiring consideration is biofilm. Bacterial biofilms are defined as clusters of bacteria surrounded by an extracellular matrix (both self-produced and environmentally derived) that protects against antimicrobials and immune mechanisms [61]. Clinical indicators of biofilm-associated infections include recalcitrance to antibiotic therapy as well as PCR-based detection of pathobionts from culture-negative specimens [62]. Biofilm has long been hypothesised to explain treatment recalcitrance in CSLD [63]; however, direct testing data are scarce. Among children with chronic wet cough, the best evidence to date is from a cross-sectional study that demonstrated biofilm in BAL from 36% of 69 children with PBB and 41% of 75 children with bronchiectasis [36]. Importantly, two categories of biofilm were observed. The first was biofilm associated with squamous epithelial cells (SEC biofilms), indicating an upper airway origin. The second was biofilm unrelated to squamous epithelial cells (non-SEC biofilms) that was hypothesised to indicate a lower airway origin. Prevalence of non-SEC biofilms was similar among children with PBB and those with bronchiectasis (odds ratio [OR] 1.24, 95%CI 0.63, 2.43), whereas SEC-biofilms were more prevalent among children with bronchiectasis (OR 2.47, 95%CI 1.20, 5.08). It was unclear whether higher SEC biofilm prevalence among children with bronchiectasis was due to sampling differences (the children with PBB were recruited from a different site) or indicative of increased microaspiration among the bronchiectasis cohort. Irrespective of the source, SEC biofilms have important implications for respiratory microbiome studies that are reliant on whole specimen DNA extracts, and, thus, risk conflating upper and lower airway microbiology. Distinguishing true lower airway microbiology from upper airway contamination has long been a challenge in the field [64,65]. The observation of SEC biofilms in BAL is an important reminder of the need for specimen quality measures to be included in all studies assessing lower airway microbiology.

It is also important to consider relationships between biofilm and neutrophilic inflammation. While neutrophils are engaged to kill bacteria and clear infection, these functions can be disabled by multiple mechanisms including cleavage of phagocytic receptors and inhibition of phagocytosis by neutrophil peptides [63]. In addition, the production of neutrophil extracellular traps (NETs) provides a substrate that supports bacterial biofilm production and persistence [61], and large biofilms incorporating NETs were observed in BAL from children with PBB and bronchiectasis [36,66]. Further research is warranted to determine whether biofilms incorporating NETs indicate a distinct microbial endotype that could be targeted with anti-NET therapies [61].

## 4. Microbiome Dynamics 

### 4.1. Interactions between Bacterial Pathobionts

Mechanistic effects of distinct bacteriome endotypes are likely underpinned by interactions between microbes. Competitive and cooperative interactions can influence pathogenic mechanisms and treatment responsiveness of the airway microbiome [67], including those between the three major pathobionts (NTHi, *S. pneumoniae*, and *M. catarrhalis*) that often co-colonise the airways of children with PBB or bronchiectasis [60,68]. Passive protection from antibiotic killing has been observed in multi-species biofilms. For example, β-lactamase-production by NTHi that was shown to protect *S. pneumoniae* from amoxicillin treatment [69]. Similarly, *M. catarrhalis* production of β-lactamase-carrying outer membrane vesicles rescued growth of β-lactamase-negative NTHi and *S. pneumoniae* from amoxicillin [70], and *S. pneumoniae* protected *M. catarrhalis* from azithromycin when grown together in biofilm [71]. In interaction studies, these pathobionts have exhibited both synergistic and competitive interactions, the outcome of which appears to be governed by environmental factors such as pH, growth phase, and arginine bioavailability [72]. *S. pneumoniae* has been shown to inhibit *M. catarrhalis* and NTHi growth via hydrogen-peroxide-mediated oxidative stress [73,74]. Conversely, *M. catarrhalis* survival and in vivo colonisation was enhanced when grown with *S. pneumoniae* in biofilm [71] and was also shown to stablilise dual-species biofilms containing NTHi and *S. pneumoniae* [75]. Further studies are needed to determine the exact nature and factors that govern the interplay between these pathobionts, particularly in the context of PBB and bronchiectasis. 

Pathobiont interactions may also affect binding to airway epithelia. NTHi, *S. pneumoniae*, and *M. catarrhalis* hijack a variety host cell receptors to facilitate airway colonisation, many of which are shared targets among these species [76]. Platelet-activating factor receptor (PAFr) on the surface of pulmonary/nasal epithelial cells and innate immune cells binds both *S. pneumoniae* and NTHi [77]. These species engage PAFr through molecular mimicry of phosphorylcholine expressed on their surfaces, an interaction that confers persistence-related properties including adherence, host cell invasion, and resistance to some host-derived antimicrobials [78,79]. Similarly, carcinoembryonic antigen-related cell adhesion molecule (CEACAM) binds *M. catarrhalis* and NTHi, promoting adhesion, host cell internalisation, and immune evasion [80]. Bacterial binding to PAFr and CEACAM upregulates expression of these receptors, promoting further bacterial adhesion [81]. Thus, rather than competition for binding sites, positive feedback generated by pathobiont binding to host receptors may prime the respiratory tract for further adhesion by species that share the same host cell receptors. NTHi appears to be central to this interaction, as it can bind and upregulate multiple receptors, and, thus, may precipitate enhanced *S. pneumoniae* and *M. catarrhalis* binding [76]. 

Other data hint at complex networks of interactions between pathobionts and the wider airway bacteriome. For example, *Haemophilus haemolyticus* was shown to protect against NTHi colonisation of respiratory epithelial cells [82,83]. Among more distantly related species, *Corynebacterium accolens* was shown to excrete a triacylglycerol lipase (LipS1) that released *S. pneumoniae*-inhibiting free fatty acids from a model host surface triacylglycerol, suggesting a host-mediated bacterial interaction [84]. Interactions with anaerobic species may also be important to treatment outcomes, as suggested by in vitro data demonstrating a clinical *Prevotella* sp. isolate produced an extended-spectrum β-lactamase that protected *Pseudomonas aeruginosa* from ceftazidime killing [85]. Although not yet assessed specifically in relation to PBB and bronchiectasis, these types of interactions highlight the potential importance of microbiome interactions to disease processes and treatment responsiveness in PBB and bronchiectasis.

### 4.2. Viral–Bacterial Interactions

Viral–bacterial co-infections have long been reported among patients with lower respiratory disease [86] and pathobiont interactions with respiratory viruses are likely to influence disease processes. For example, influenza infection had long-term impacts on airway bacteriomes and host responses to bacterial infections in a mouse model [87], and HRV infection in infants was associated with NTHi and *S. pneumoniae* overgrowth and persistence [88,89]. Several respiratory viruses upregulate bacterial binding sites on host cells, which may explain the significantly higher pathobiont loads in the upper respiratory tract of children when a virus is co-detected [90,91]. Influenza and RSV are well-recognised for their interactions with bacteria in the respiratory tract and were shown to mediate NTHi, *S. pneumoniae* and *M. catarrhalis* adherence to epithelial cells [92], and to cause epithelial damage that exposes basement membranes [93]. Additionally, in vitro studies demonstrated RSV can upregulate pulmonary epithelial cell line expression of PAFr [94]. Similarly, HRV was shown to augment expression of CEACAM and intracellular adhesion molecule (ICAM-1) (both of which bind NTHi and *M. catarrhalis*) [81,95], whereas enhanced *S. pneumoniae* adherence and invasion was observed when epithelial cells were infected by adenovirus, RSV, HRV, or influenza [96,97]. Respiratory viruses were also shown to promote bacterial growth and persistence indirectly through immune-mediated mechanisms, with influenza-induced depletion of pulmonary macrophages resulting in *S. pneumoniae* overgrowth [98]. 

### 4.3. The Gut–Lung Axis

Airway environmental factors are critical to shaping the lower respiratory microbiome, including systemic immune factors that exert selective pressure. It is increasingly recognised that systemic factors induced by host–microbial interactions in the gut microbiome may influence the respiratory microbiome; a concept referred to as the gut–lung axis. This axis is a complex communication pathway between the gastrointestinal and respiratory systems that is implicated in modulation of homeostatic and disease processes via interactions between microbes and host immune cells [99]. Initial evidence for the existence of a gut–lung axis was from studies demonstrating indirect associations between the gut and respiratory microbiomes. For example, dietary modifications in newborns were associated with lung microbiome composition [100], and a high prevalence of pulmonary disease was observed among patients with gastrointestinal disease [101,102,103]. 

The importance of the gut–lung axis has become more evident since identification of gut microbe-derived metabolites that can mediate host immunity [99]. Evidence to date suggests the primary direction of crosstalk is from the gut to the lungs [104,105]; however, translocation and migration of metabolites, immune mediators, and microbial fragments via the lymphatic system can also occur from the lungs to the gut [106]. Furthermore, ecological links and physiological mechanisms have demonstrated coordinated development of the gut and lung microbiomes [107], such that the microbial composition at one site is highly predictive of composition at the other, irrespective of underlying differences in the taxa present at each site [108]. Animal models suggest plausible mechanisms for the distal effects of the gut microbiome on lung infections [109]. For example, antibiotic-driven reductions in the density and diversity of commensal gut bacteria in mice were shown to increase susceptibility to pneumococcal pneumonia due to a failure of gut dendritic cells (DCs) to initiate the trafficking of anti-bacterial innate lymphoid cells from the gut to the lung [110]. Oral probiotics (e.g., *Lactobacillus*, *Bifidobacterium*) can reverse artificially induced gut dysbiosis in mice, increasing short-chain fatty acid (SCFA) production and protecting against RSV challenge [111]. A recent mouse study demonstrated a causal pathway (involving propionate and the growth factor Flt3L) in which neonatal gut dysbiosis perturbed DC maturation and trafficking to the lung, increasing ALRI susceptibility [112]. As such, understanding of the gut–lung axis opens new therapeutic opportunities (e.g., probiotics or microbial metabolites) for promoting a health-associated gut microbiome and, in turn, resilience against respiratory infections.

The gut–lung axis has been implicated in several respiratory diseases, including CF, asthma, chronic obstructive pulmonary disease (COPD), and adult bronchiectasis [113,114,115,116,117]. It is expected that the gut–lung axis will also influence PBB and bronchiectasis in children, but there are currently no data to report. Deeper understanding of the gut–lung axis development in infants may be crucial, given the young age of children presenting with PBB and bronchiectasis (commonly < 3 years) and the importance of early life microbial colonisation to systemic immune development. Early life microbial dysbiosis can have long lasting health consequences, even after the microbiome stabilises [118,119], as evidenced by reports showing that reduced abundance of several common gut commensal microbes is associated with increased risk of atopy and asthma [120,121]. It remains to be determined whether similar relationships influence paediatric PBB and bronchiectasis risk.

## 5. Moving beyond Amplicon Sequencing Data 

### 5.1. Multiomics

Multiomics is used to describe studies that integrate multiple ‘omics data types into a single analysis [122], and will likely be essential to realising nuanced respiratory endotypes. There is a dearth of integrated microbiome, transcriptome, and/or metabolome analyses in PBB and paediatric bronchiectasis; however, progress has been made in other respiratory contexts [122]. Ideally, metagenomic data (describing the composition and functional potential of airway communities) should be used; however, PBB and paediatric bronchiectasis metagenome data are lacking. Furthermore, as noted above, excess human DNA in respiratory specimens (particularly where neutrophilic influx has occurred) can dominate metagenomic data. While microbial characterisation is improved by selective human DNA depletion [123,124,125], high proportions of host data may still remain (>60% of reads) [38]. Extra deep sequencing (whereby the amount of sequence data generated is increased to support improved microbial characterisation) can mitigate some host DNA effects, but can become cost prohibitive, and may not yield sufficient microbial data to characterise the populations beyond basic taxonomic classification [126]. Despite the limitations, metagenomic data have been generated successfully from adult bronchiectasis sputum specimens [23,127]. Advances in multiomic analyses of PBB and bronchiectasis are expected in the coming years.

### 5.2. Diagnostic Translation of ‘Omics Data

Diagnostic testing of respiratory microbiology remains broadly reliant on culture and/or PCR-based tests; however, culture can lack sensitivity and PCR-based methods target only a small number of species [28,60,128]. The availability of real-time DNA sequencing technology heralds a new era of diagnostic applications [129]; however, translation into microbiome-based tests has yet to be realised. As discussed above, diagnostic respiratory endotyping is likely to require integration of microbial and host response data (either transcriptomic data or targeted quantification of immune biomarkers). Additionally, detailed biological profiling may be supported by metabolomic analyses that measure small molecules in biological samples at scales ranging from intracellular components through to detection of whole organisms [130]. Promising results have been obtained from metabolomic studies of adult respiratory specimens [122]; however, data from children with PBB and bronchiectasis are lacking.

Advances in non-invasive airway sampling methods will also be critical to progressing paediatric respiratory diagnostics, particularly regarding PBB and bronchiectasis affecting infants and young children who do not spontaneously expectorate. BAL and protected brushes have been used to sample the lower airways of children with PBB and bronchiectasis [28,30]; however, these techniques require general anaesthesia, and, thus, do not support longitudinal analyses. Induced sputum is a non-invasive alternative; however, careful analysis and data interpretation is needed due to the risk of salivary contamination [65]. Metabolomic analysis of breath shows promise as an emerging non-invasive sampling method. One particular approach is to measure the composition of molecules in breath (both host and microbial) and test for the presence of molecular biomarkers predictive of disease status [131]. It is not yet clear whether breath-based metabolomic profiling could be used to identify PBB and bronchiectasis endotypes; however, progress has been made in defining metabolomic markers of taxa present in the airway microbiome. One promising line of research is volatilomics—a technique that profiles the volatile molecular fraction of the metabolome. Species-specific volatile markers have been identified from NTHi, *S. pneumoniae*, and *M. catarrhalis* cultures [132,133,134], including serotype-specific pneumococcal markers [133]. Culture-based studies have also identified volatile molecules that distinguish viral (including RSV and adenovirus) from bacterial cultures (including NTHi and *M. catarrhalis*) [134]. 

It is not yet clear whether culture-based findings will translate to breath content; however, progress has been made in establishing breath-based detection of volatile markers predictive of *P. aeruginosa* and *Staphylococcus aureus* infection among CF patients. For example, a panel of nine breath volatile molecules distinguished CF patients with and without *S. aureus* infection [135]. Another study demonstrated hydrogen cyanide was present at elevated concentrations in the breath of CF patients who had *P. aeruginosa* infections [136]. Moreover, a combination of 16 volatile molecules from breath was found to discriminate *P. aeruginosa*-positive from *P. aeruginosa*-negative CF patients [137]. Headspace analysis of respiratory specimens can also be useful. For example, sputum headspace analysis demonstrated 2-nonanone as a distinctive marker of *P. aeruginosa* infection in CF and non-CF adult bronchiectasis patients compared to *P. aeruginosa*-negative patients [138]. Importantly, among CF patients with *P. aeruginosa* and *S. aureus* co-infection, 6 to 11 volatile molecules in BAL fluid headspace were shown to discriminate these species [139]. Realisation of breath-based volatilomic lower airway diagnostics is not without challenges though, including the risk of upper airway pathobiont colonisation potentially confounding attempts to diagnose lower airway infection. Careful assessment for the presence of upper respiratory secretions in BAL and sputum will be essential where these specimen types are used as the gold-standard comparator in development of breath-based diagnostics.

## 6. Emerging Therapeutic Approaches

Current treatments for children with PBB, CSLD, and bronchiectasis are largely limited to antibiotics (either oral or intravenous), with recommended therapies differing depending on clinical presentation, whether culture-based pathobiont data are available, and whether the patient is colonised with *P. aeruginosa* [12]. Long-term (>6 months) low-dose macrolide therapy is recommended for patients with frequent exacerbations (≥3 episodes requiring antibiotics in 12 months) and no contraindications [12], both to clear bacterial agents and to leverage the immunomodulatory properties of these drugs [140]. However, recurrent airway exacerbations among children with PBB and bronchiectasis, even after receiving recommended therapies, indicate a need for new therapeutic approaches. Therapeutic approaches emerging from improved understanding of the respiratory microbiome include anti-biofilm, phage, and probiotic therapies.

### 6.1. Anti-Biofilm Therapies

Bacteria living in biofilms can survive antibiotic treatment, even when planktonic cells of the same strain are susceptible [141,142]. As such, anti-biofilm therapy may be required to clear persistent infections. Anti-biofilm agents are not included in current PBB and bronchiectasis treatment guidelines; however, macrolide antibiotics can impair bacterial biofilm synthesis [143]. Indeed, in vitro studies have demonstrated azithromycin can decrease formation of and disrupt established NTHi biofilms [142,144]. 

Several other anti-biofilm therapies are under development; however, most have been assessed against *P. aeruginosa* and efficacy against biofilms produced by the major paediatric respiratory pathobionts is unknown. Examples include aerosolised powders that deliver high antibiotic concentrations that can penetrate the biofilm matrix and may also target intracellular bacteria [145]. Liposomal formulations of these agents were shown to enhance biofilm penetration in animal models [146], and a ciprofloxacin–mannitol/lactose nanoplex improved mucus permeability and achieved superior efficacy against *P. aeruginosa* PAO1 biofilms compared to native ciprofloxacin [147]. However, despite the increased anti-biofilm efficacy, ciprofloxacin nanoplexes were also observed to induce cytotoxic effects where the antibiotic concentration became highly localised [148]. Other research has proposed re-purposing of N-acetyl cysteine (NAC; a mucolytic agent with antioxidant, anti-inflammatory, and antibacterial activity) as an anti-biofilm agent, which has been shown to inhibit formation of and disrupt established biofilms [149,150]. Quorum-sensing inhibiting quercetin–chitosan nanoparticles were also shown to be effective against *P. aeruginosa* biofilms in vitro; however, clinical potential of this agent remains to be determined [151]. Another line of research is assessing inhaled nitrite for activity against *P. aeruginosa* biofilms. In vitro, sodium nitrite prevented 99% of *P. aeruginosa* biofilm growth when tested on primary human airway epithelial cells; efficacy was enhanced when combined with colistimethate [152].

Drugs that are used currently to treat other chronic biofilm-associated infections also require consideration. One such therapy is dornase alfa (Pulmozyme^®^), a recombinant human deoxyribonuclease that is used as a mucolytic agent in treating CF. Dornase alfa is not used currently in bronchiectasis after an earlier trial demonstrated rates of FEV1 decline and pulmonary exacerbations both increased among stable adult bronchiectasis patients who received this treatment compared to the placebo-treated control group [153]. An important limitation of this study was that dornase alfa was administered without adjunct antibiotic therapy. As the dornase alfa mechanism of action is expected to release planktonic bacteria from biofilm and NETs, treatment without adjunct antibiotic therapy risks triggering of an active infection among stable patients with otherwise quiescent infections. In vitro assays assessing biofilm degradation and antimicrobial susceptibility following dornase alfa treatment are warranted prior to further clinical trials.

Other novel approaches targeting extracellular DNA (eDNA) in the biofilm matrix are under development. eDNA shields bacteria from hostile environments, allowing a sessile metabolic state and enables interactions between bacteria co-residing within biofilms [154]. eDNA targeting leads to biofilm collapse and enables bacterial eradication. Both vaccine-based prevention and direct therapeutic strategies targeting the DNABII protein integration host factor (IHF) have been described [155]. These approaches, specifically targeting IHF through immunisation or through the use of a polyclonal antiserum directed against IHF, have demonstrated high efficacy against NTHi, *S. pneumoniae*, and *M. catarrhalis* biofilms in vitro and in a chinchilla model of NTHi-induced otitis media [155,156,157]. Importantly, for a short period, bacteria released from biofilm by these therapies were observed to be more sensitive to antibiotics than the planktonic cells that initially formed the biofilms, suggesting a critical point where bacteria may be more readily cleared from the host [156]. It is unclear whether similar efficacy could be achieved in chronically infected lungs; however, as with dornase alfa, it is likely that adjunct antibiotic therapy would be essential to any clinical use of these agents.

### 6.2. Phage Therapy

Phage therapy relies on the use of bacteriophage viruses that infect bacteria. A number of phage characteristics make them amenable to potential therapeutic use: (1) high species specificity, thereby enabling pathobiont targeting while minimising microbiome disruption [158]; (2) ability to penetrate and disrupt biofilms [159]; (3) ability to kill antibiotic-resistant persister cells [160]; (4) ability to re-sensitise bacteria to existing antibiotics [161]; and (5) generally good tolerability in clinical trials [162,163]. Clinical trials of phage therapy for pulmonary infections are scarce [164,165], with no trials specific to PBB or bronchiectasis. However, phage therapy candidates are being investigated against common respiratory pathobionts, including NTHi, *S. pneumoniae*, *M. catarrhalis*, and *P. aeruginosa* [13,166].

To date, most phage therapy candidates have been assessed against *P. aeruginosa*, with a mixture of clinical trials [163,165], single compassionate-use cases [167,168,169,170], and in vitro model studies [171,172,173,174]. The single completed phase I/II clinical trial included chronic otitis media patients and found phage therapy yielded the best clinical outcomes against antibiotic resistant *P. aeruginosa* infections, significantly lowered bacterial loads, and did not elicit adverse events [163]. In the cases of compassionate use, all reported either clinical improvement [167,169] or complete eradication [168,170] after phage therapy. Of note, in the two cases with complete eradication, phage therapy was combined with antibiotics or immunoglobulin therapy, highlighting phage therapy’s potential for synergistic effects. In biofilm studies, combination therapy holds promise but should be approached cautiously, as both synergistic and antagonistic effects have also been described between certain classes of antibiotics, albeit uncommon [174,175]. In vitro evidence generally supports the outcomes seen in patients, with treatments in murine chronic lung infection models completely clearing [174] or prophylactically preventing [171] *P. aeruginosa* infections, while also demonstrating biofilm penetration and reduction [174,176]. Interestingly, a genetically engineered super-infective phage induced a small-colony variant of *P. aeruginosa* that eliminated its ability to disseminate systematically in a murine wound model [173]. Among the major PBB and bronchiectasis pathobionts, only one limited trial has been conducted, which shows phage-based rescue of mice infected with NTHi and *M. catarrhalis* [177]. 

Despite these promising results, variable storage stability and the need to screen individual patient bacterial strains against phage panels prior to administration is likely to limit broad uptake of phage therapy [178,179]. To overcome this, an offshoot of phage therapy relying on phage-derived enzymes (PDEs) is under investigation [178]. These enzymes are used natively by phages to digest holes in the bacterial peptidoglycan cell wall in order to enable either entry into the cell (e.g., pyocins and depolymerases) or exit after infective replication (endolysins) [162,180,181]. PDE’s have broad specificity, can penetrate biofilm and have capacity to kill metabolically inactive bacterial cells [162,182]. Additionally, PDEs have rapid killing action, with 4-log reductions of *S. pneumoniae* bacterial loads observed in as little as 15 min [183]. Similar to phage therapy, PDEs show enhanced or synergistic activity when combined with other PDEs or antibiotics [184,185]. Unlike antibiotics and phage therapy, however, risk of PDE resistance development is low [182], presumably due in part to their rapid mode of action and the conserved nature of their peptidoglycan targets. Challenges remain for the development of PDEs into frontline therapeutics, with a primary limitation being limited endolysin penetration of Gram-negative bacterial outer membranes that prevents access to the peptidoglycan cell wall [162]. Solutions to this issue are emerging, with identification of endolysins that have native external activity against Gram-negative bacteria [186], and engineered endolysins [187] that show promising results. Finally, due to their biological nature and recent (re)emergence, both phage and PDE candidate therapies are facing uncertainty with outdated and changing regulatory and intellectual property hurdles [162,178,188]; however, the future is promising for these novel antimicrobials, particularly for PDEs that are more amenable to contemporary manufacturing, regulation, and clinical application than native phage [178].

### 6.3. Probiotics

Probiotics are under investigation for their potential to passively prevent ALRI in infants and young children. Early onset infant pneumonia and/or recurrent ALRIs are arguably the leading modifiable paediatric CSLD risk factor [189,190,191]. Early onset ALRIs are almost universal among populations with high paediatric CSLD burdens [189,192,193]. Therapies that prevent or delay early onset ALRI have potential to achieve sustainable improvements in lung health across the life course.

Probiotics can promote resilience against respiratory infection by modifying host immune responses, either directly or via the gut–lung axis. First microbial colonisers of the neonatal gut are pivotal to immune development and future disease risk [194]. Generally, facultative anaerobes (e.g., *Lactobacillaceae*) are the first gut colonisers followed by true anaerobes (e.g., *Bifidobacteriaceae*) [195]. Colonisation by these beneficial, milk-digesting microbes is impacted by multiple factors, including antibiotic exposure, birth mode, diet, environment, and genetics [195,196,197,198,199]. Additionally, while the immune system at birth is T-helper (Th) 2 cell biased [110], the lack of effective Th1 responses renders infants vulnerable to infection. Appropriate and early microbial exposures are essential to development of a balanced Th1/Th2 response. Moreover, commensal microbes (e.g., *Lactobacillus*, *Bifidobacterium*) can out-compete gut pathogens, enhance barrier integrity, and intricately modulate innate and adaptive immune responses [200,201]. The ideal window for microbiome-induced immune priming is the first 100 days of life [202]. 

Numerous randomised controlled trials (RCTs) have tested whether oral probiotics prevent ALRI [203,204]. Despite substantial heterogeneity between studies, meta-analyses found 60% of 83 RCTs showed probiotics significantly reduced ALRIs, with effect sizes ranging from OR 0.53 (95%CI 0.37, 0.76) [204] to 0.84 (95%CI 0.74, 0.95) [203]. Importantly, a Cochrane review [204] found probiotics halved ALRI rates (47% reduction vs placebo) and significantly reduced antibiotic use, ALRI duration, and school absenteeism. However, confirmatory studies remain necessary due to the underlying heterogeneity between published trials. Meta-analysis of seven RCTs published up to June 2023 that tested probiotics (vs. placebo) for prevention of ALRI in neonates or infants (<3 months of age) [205,206,207,208,209,210,211] showed probiotics were better than placebo in reducing ALRI risk (RR 0.79; 95%CI 0.64, 0.98). The most common probiotic genera tested were *Lactobacillus* and *Bifidobacterium*. 

While these data suggest oral probiotic effects improve respiratory health via the gut–lung axis, animal model data suggest the lung bacteriome correlates better with respiratory immunity [212,213]. Delivery of probiotics and/or microbial metabolites (e.g., SCFAs) directly onto the respiratory mucosa offers a novel approach to managing lung infection and inflammation. As the respiratory bacteriome co-evolves with the immune system and predicts infection risk [44,214], respiratory probiotic interventions are likely to provide the most benefit if delivered early in life, as with gut-based therapies. To date, respiratory probiotics have been investigated primarily in vitro and in animal models. Human data are sparse; however, several lines of enquiry suggest clinical utility. For example, several taxa have probiotic activity against respiratory viruses, including *Enterococcus* and *Lactobacillus* species that can inhibit influenza entry into host cells [215,216,217], secrete anti-viral metabolites [218], and enhance innate and adaptive immune responses [219]. These taxa also protected against pneumococcal, influenza, and RSV infection in mice [220,221]. Likewise, intranasal administration of *Bifidobacterium longum* reduced influenza viral load in a mouse model [222]. Other studies show oropharyngeal *H. haemolyticus* carriage correlates with reduced NTHi prevalence and density [223], with anti-NTHi activity also demonstrated in vitro [83]. Taken together, these data indicate potential therapeutic utility of respiratory-administered probiotics; however, progression of respiratory probiotics into clinical trials first requires improved understanding of the key microbes and metabolites, delivery methods, timing, efficacy in humans, effect duration, safety, and generalisability of use (for example, among children or those with existing chronic lung disease). Individual-level clinical and microbial endotype data are expected to be essential to guiding best practice.

Finally, bacterial extracts are also under investigation in CSLD. To date, there is a single published RCT assessing OM-85 (Broncho-vaxom^®^, Vifor Pharma Pharmaceutics) that contains extracts of eight bacteria commonly implicated in respiratory infections [224]. This study found OM-85 had no significant effect on the number of exacerbations experienced by adult bronchiectasis patients during the one-year intervention period, or during follow-up [224]. We are unaware of any published paediatric studies.

## 7. Summary

**Figure 3:**
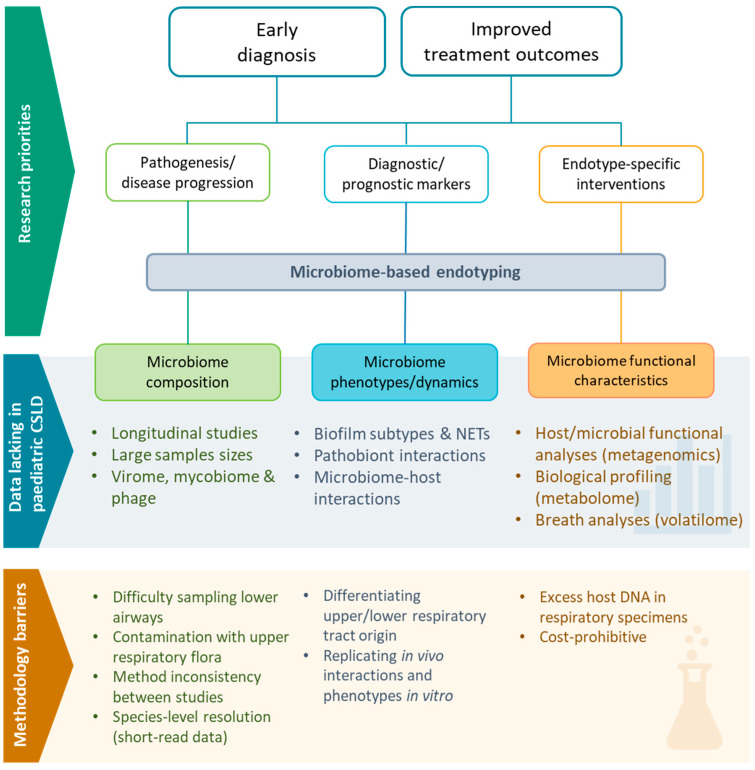
Summary of the discussed research priorities, remaining knowledge, and methodological barriers associated with developing microbiome-based endotyping in paediatric CSLD.

Understanding of the complex microbial and host factors underlying PBB and bronchiectasis continues to grow, but much remains to be determined (Figure 3). To date, challenges inherent to sampling the lower airways of young children have limited understanding of the paediatric PBB and bronchiectasis continuum relative to adult disease. Large gaps remain in the understanding of the PBB and bronchiectasis bacteriome, wider microbiome (including gut–lung interactions), and host responses underlying disease establishment, progression, and treatment responses, with few studies published to date. Addressing these gaps is critical to realising improved strategies for halting disease progression in paediatric contexts, particularly as findings from adult studies may not be generalisable to children [60]. 

Despite the limited available data, PBB and bronchiectasis bacteriome studies to date are suggestive of distinct microbial endotypes [28,29,31], like those reported among adult bronchiectasis patients [22]; however, reliance on short-read 16S rRNA gene sequences limits taxonomic certainty. Shotgun metagenomic data is required to resolve species-level identification, support virome characterisation, and to enable functional assessment of microbial communities (including resistome analyses) [225]. In the event that low biomass and excess human DNA hamper efforts to determine PBB and paediatric bronchiectasis metagenomes, full-length 16S rRNA gene amplicon sequencing should be considered to support species-level compositional analyses [226]. Likewise, transcriptomic and metabolomic analyses remain a priority in order to progress establishment of clinically meaningful endotype definitions and to support new diagnostic applications. 

Advances in diagnostic tests will be critical to improving endotype definition and translating findings into feasible clinical applications. This includes a need for imaging-based studies to complement ‘omics-based data, particularly in regards to biofilm and NETs, which both may indicate clinically important endotypes [36]. Biofilm diagnostic tests suited to clinical laboratory settings are lacking; this is an important gap that needs to be addressed as a research priority. Additionally, specimen quality assessments need higher consideration to ensure that impacts from oral contamination in respiratory specimens is minimised in endotyping efforts. Despite the availability of simple staining methods for assessing oral secretion contamination [64,65], specimen quality assessments are rarely (if ever) considered in respiratory microbiome studies.

Technological advances supporting breath-based molecular profiling open substantial new opportunities for respiratory diagnostic test development. While metabolomic data to date are promising, substantial gaps remain to be addressed. This is particularly the case in paediatric contexts. Importantly, it remains to be determined whether (or how) nuanced differentiation of molecular signals from upper and lower airways in paediatric breath samples could be achieved; however, this limitation equally affects sputum, induced sputum, and BAL-based measures of airway microbiology. Despite current limitations, major advances in metabolomic profiling of paediatric respiratory samples, including breath, are expected in the coming years, particularly given the establishment of global initiatives focused on advancing breath-based molecular profiling (e.g., the Human Breath Atlas; https://humanbreathatlas.com/ accessed on 26 October 2023).

Translation of microbiome knowledge to new therapeutics is also expected in the coming years, with increasing evidence describing the efficacy of novel anti-biofilm-, phage-, and probiotic-based therapies. Despite recent advances and promising clinical studies, more data specific to PBB and paediatric bronchiectasis are needed. Furthermore, current in vitro data are weighted towards model *P. aeruginosa* strains, and, thus, it remains to be determined whether similar efficacy will be achieved against clinical strains of the major paediatric respiratory pathobionts. Mechanistic studies elucidating interactions between pathobiont species and the wider airway microbiome are growing, but considerably more research effort is needed to understand the complexity of microbe–microbe and host–microbe interactions (including gut–lung interactions) that may influence PBB and bronchiectasis pathogenesis and treatment responsiveness. Much in vitro data will be needed before emerging therapeutic research directions can be translated into clinical trials.

## 8. Conclusions

The lower airway microbiome among children with chronic wet cough remains less understood than the respiratory microbiome among adults with chronic lung diseases. Despite the limitations of current data, advances to date point towards emerging opportunities for expanded understanding of microbial and host factors that could be used to define clinically important endotypes. Multiple promising lines of enquiry are focused on development of new diagnostic and therapeutic approaches. It is critical that future chronic airway disease research include paediatric contexts to ensure that the benefits of progress being made in adult contexts also extend to children with PBB and bronchiectasis.

## Figures and Tables

**Table 1:** Clinical definitions of PBB, CSLD, and bronchiectasis.

Condition	Clinical Definition
PBB	Isolated chronic wet or productive cough without signs of another cause which typically resolves following a 2–4 week course of appropriate oral antibiotics [2].
CSLD	Recurrent wet or productive cough episodes (≥3 per year), each lasting for >4 weeks, with or without other features (for example, exertional dyspnoea, symptoms of airway hyperresponiveness, recurrent chest infections, growth failure, clubbing, hyperinflation or chest wall deformity [3].
BE	Symptoms and/or signs outlined for CLSD as well as characteristic radiographic features on chest high-resolution computed tomography (such as bronchial lumen dilation and wall thickening) [3].
